# Determining the optimum morphology in high-performance polymer-fullerene organic photovoltaic cells

**DOI:** 10.1038/ncomms3867

**Published:** 2013-12-17

**Authors:** Gordon J. Hedley, Alexander J. Ward, Alexander Alekseev, Calvyn T. Howells, Emiliano R. Martins, Luis A. Serrano, Graeme Cooke, Arvydas Ruseckas, Ifor D. W. Samuel

**Affiliations:** 1 Organic Semiconductor Centre, SUPA, School of Physics and Astronomy, University of St Andrews, North Haugh, St Andrews, Fife KY16 9SS, UK; 2 Materials and Condensed Matter Physics, SUPA, School of Physics and Astronomy, University of Glasgow, Glasgow G12 8QQ, UK; 3 Glasgow Centre for Physical Organic Chemistry, WestCHEM, School of Chemistry, University of Glasgow, Glasgow G12 8QQ, UK

## Abstract

The morphology of bulk heterojunction organic photovoltaic cells controls many of the performance characteristics of devices. However, measuring this morphology is challenging because of the small length-scales and low contrast between organic materials. Here we use nanoscale photocurrent mapping, ultrafast fluorescence and exciton diffusion to observe the detailed morphology of a high-performance blend of PTB7:PC_71_BM. We show that optimized blends consist of elongated fullerene-rich and polymer-rich fibre-like domains, which are 10–50 nm wide and 200–400 nm long. These elongated domains provide a concentration gradient for directional charge diffusion that helps in the extraction of charge pairs with 80% efficiency. In contrast, blends with agglomerated fullerene domains show a much lower efficiency of charge extraction of ~45%, which is attributed to poor electron and hole transport. Our results show that the formation of narrow and elongated domains is desirable for efficient bulk heterojunction solar cells.

## Abstract (web summary)

The morphology of organic solar cells is crucial to their performance but is difficult to measure. Using a variety of probes, Hedley *et al.* map the morphology of polymer-fullerene solar cells and find that elongated fibre-like polymer- and fullerene-rich domains are desirable for high performance.

## Graphical abstract

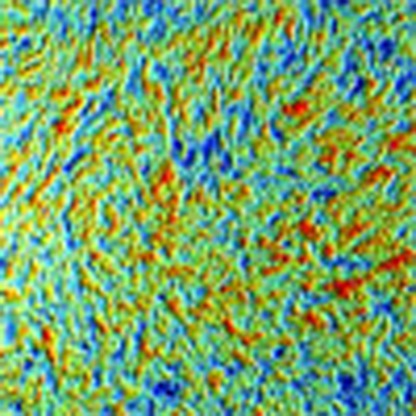

In organic photovoltaic cells (OPVs), the highest power conversion efficiencies are achieved in bulk heterojunction (BHJ) devices in which the active layer is made by blending electron donor and acceptor materials^1^,^2^,^3^,^4^. Light absorption in each of these materials generates excitons that have to diffuse to an interface with the other material, where there is an offset in energy that can split them into electron–hole pairs. These pairs then have to overcome Coulombic attraction in order to dissociate into free-charge carriers and to be extracted to the corresponding electrodes via percolation pathways. Critical to all of these processes is the morphology of the blend—how the two materials mix together or segregate. It is a pressing challenge in the science of OPV materials and devices to be able to determine the optimum morphology of blends^5^,^6^,^7^,^8^,^9^,^10^,^11^,^12^,^13^,^14^,^15^.

Recently, single layer OPVs with a record power conversion efficiency of 9.2% were reported^16^ using a blend of the fullerene derivative [6,6]-phenyl-C_71_-butyric acid methyl ester (PC_71_BM) and a polymer with alternating units of thieno[3,4–b]thiophene and benzodithiophene, commonly denoted as PTB7 (chemical structures shown in Fig. 1a) and so consequently this system is of great interest. In this Article, we investigate the nanoscale morphology of such blends, their ultrafast photophysics and performance in devices. We use advanced scanning probe techniques and also exciton diffusion in PC_71_BM as a nanoscale ruler to determine the domain sizes. We investigate the exciton dissociation dynamics with a femtosecond time resolution, which allows us to get information on the domain composition. Blends were prepared with and without the high boiling point additive diiodooctane (DIO). Addition of DIO improves the device’s internal quantum efficiencies by a factor of 2, which we determine is mainly because of improved charge extraction. We show that blends prepared with DIO have a fibre-like morphology of polymer-rich and fullerene-rich domains. We suggest that a concentration gradient between these domains helps in the dissociation of geminate pairs by charge diffusion and that charge extraction is enhanced with a fibrous morphology. We conclude that a fibrous morphology of polymer-rich and fullerene-rich domains is highly advantageous for OPVs.

## Results

### Photovoltaic response

**Figure 1:**
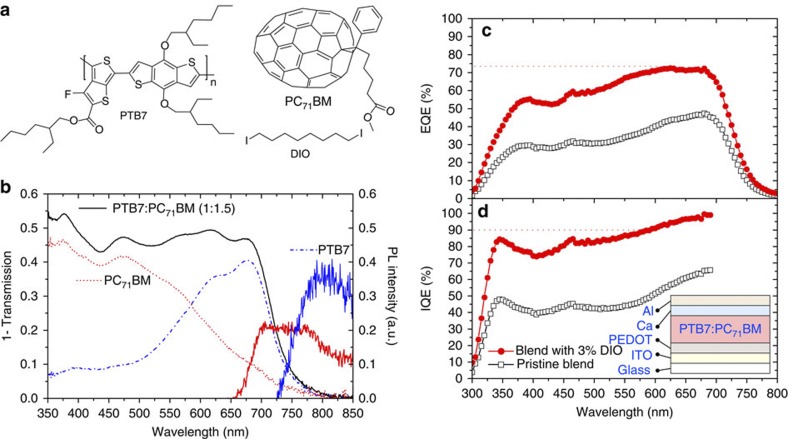
Chemical structures and device characteristics. (**a**) Chemical structures of PTB7, PC_71_BM and the additive DIO. (**b**) Absorption spectra of PTB7 (blue dot-dashed line) and PC_71_BM (red dotted line) films and a 1:1.5 blend film of the two materials (black line). The PL spectra of PC_71_BM (red solid line) and PTB7 (blue solid line) are shown, paired to the right-hand *y* axis. Solar cells prepared from this blend with and without additive give devices with external quantum efficiencies (**c**) and internal quantum efficiencies (**d**) as shown. The IQE >700 nm has been omitted owing to the falling edge of absorption at these wavelengths. The device performance parameters are as follows. Pristine blend: power conversion efficiency=3.15%, *V*_OC_=0.719 V, *J*_SC_=−9.17 mA cm^−2^ and fill factor=0.478. Blend with 3% DIO: power conversion efficiency=6.34%, *V*_OC_=0.703 V, *J*_SC_=−15.45 mA cm^−2^ and fill factor=0.584. The device architecture is inset in **d** with the fabrication as described in the Methods.

Absorption spectra of PTB7 and PC_71_BM films as well as the 1:1.5 blend of the two are shown in Fig. 1b, along with the photoluminescence (PL) spectra of PC_71_BM and PTB7. OPVs were prepared with and without the additive DIO as described in Methods and the device characteristics are shown in Fig. 1c,d and current–voltage curves in Supplementary Fig. S1. The electrode structures were not optimized for the highest possible power conversion efficiencies. It should be noted that the efficiency of our device is comparable to the reported value of 7.4% with the same architecture^7^. The world record 9.2% efficient device was fabricated in an inverted geometry with optimized electrode work-functions that increased the photocurrent. However, the active layer deposition process of the record cell (blend ratio, solvent, use of additive, spin-coating speed) was identical to the deposition parameters we have used to make films. Thus, the morphology and photophysics of our blend films, and the conclusions we deduce, are transferable to devices with the highest reported efficiencies. The external quantum efficiency (EQE) increased by a factor of 2 in devices prepared with DIO and is consistent with previous reports^7^,^8^. The internal quantum efficiency (IQE)—corrected for the number of photons absorbed in the active layer—is determined by optical modelling of the device stack and also increases by a factor of 2 in devices prepared with DIO. The IQE is >90% with DIO in the polymer absorption region and is slightly lower in the region of fullerene absorption.

### Blends prepared without additive

**Figure 2:**
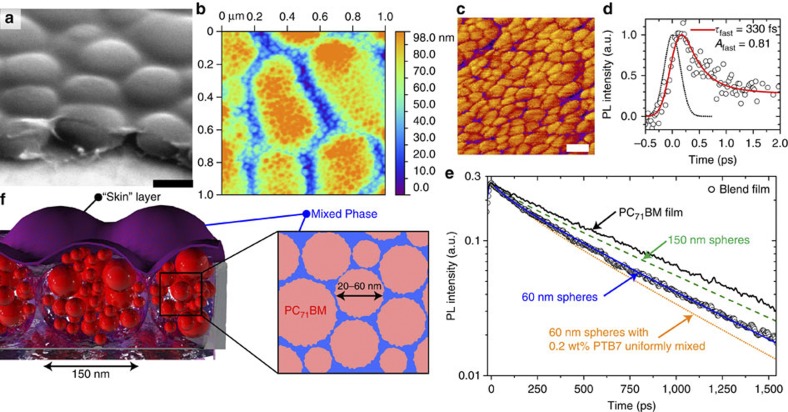
Results of PTB7:PC_71_BM blends prepared without additive. (**a**) SEM image of the blend viewed from an angle of 52° with a score made across the film showing large domains and a skin of material on top, the black scale bar is 250 nm. Removing the skin by plasma-ashing the topography of the domains measured with AFM is shown in **b**, and in phase mode in **c**, indicating that the large domains consist of smaller spheres of material, where the white scale bar is 50 nm. The time-resolved photoluminescence in the blend is presented on short (**d**) and long (**e**) timescales, with the blend decay shown as open circles. In **d**, a best fit to the decay (red line) with a time constant of 330 fs and a pre-exponential factor of 0.81 is found. The slower part is shown in **e**, indicating that the decay is faster in the blend when compared with a film of PC_71_BM (black line). The blue line is the best fit from the model for single 60-nm spheres of pure fullerene. The green dashed line is a simulated decay if the pure fullerene spheres were 150 nm, while the orange dotted line is a simulated decay if the spheres were 60 nm of fullerene with 0.2 wt% of PTB7 mixed inside them; neither fits the experimental data. (**f**) Schematic of morphology, showing large fullerene-rich domains comprised of small spheres of pure PC_71_BM surrounded by a PTB7-rich mixed phase. A finely mixed phase of the two materials sits between the large domains and also forms a skin on top of the film.

We have combined time-resolved PL (TRL) and atomic force microscopy (AFM) to provide complementary information on phase separation of the fullerene and polymer. Previous studies reported large domains of fullerene of 100–200 nm in diameter^7^,^8^,^10^ in blends prepared without additive. With scanning electron microscopy (SEM) we indeed see large domains (Fig. 2a). To extract more information, we made a score through the film to enable the profile of the domains to be imaged. Visible in the same SEM image is a top ‘skin’ layer of material that can be clearly seen to have been pulled back from the domains at the score, enabling us to observe the material beneath. Then we removed 30–40 nm of material by plasma-ashing to take away the top skin and measured the AFM topography (Fig. 2b), which shows the large 100–200 nm diameter domains but with fine structure inside them. In an AFM-phase image (Fig. 2c), the fine structure is better resolved and shows small sphere-like objects of 20–60 nm diameter. We have then employed TRL measurements to learn about the composition of the blend.

TRL intensity from PC_71_BM is proportional to the exciton population in the fullerene, and thus quenching of PC_71_BM emission by PTB7 is able to tell us about the segregation of molecules. Neat films of PTB7 are fluorescent; however, it is important to note that even with selective excitation of PTB7 in the blend (*λ*_ex_=670 nm) no polymer fluorescence was observed, even at timescales as short as 2 ps; thus with 400 nm excitation we look at solely PC_71_BM emission. Previously, Kazaoui *et al.*^17^ have reported that with high-energy photoexcitation it is possible to excite intermolecular charge transfer states in neat films of C_70_, directly producing charge pairs from an absorbed photon. To confirm that we were not generating significant amounts of charge pairs directly, we measured the PL excitation spectra and compared it with the absorption spectrum of the film (see Supplementary Fig. S2) and find that at 400 nm, ~90% of the absorbed photons produce excitons, with ~10% producing charge pairs. The discrepancy between absorbed photons and emitted photons only becomes significant below 375 nm. The decay of PL is observed on femtosecond (Fig. 2d) and picosecond–nanosecond (Fig. 2e) timescales. Decay of the PL in the blend is faster than in films of only PC_71_BM (Fig. 2e), indicating dynamic quenching by the polymer. The ultrafast decay of PC_71_BM emission in Fig. 2d indicates a 330-fs quenching with 0.8 amplitude of fullerene excitons by PTB7. This can occur by hole transfer to PTB7 or resonant energy transfer (RET) to the polymer. The Förster radius for RET from PC_71_BM to PTB7 was calculated to be 2.17 nm from the spectral overlap as described in the Methods. From this value we conclude that the fast quenching has to be short ranged in nature (<10 nm) and is consistent with a large number of small spheres of fullerene, presenting a large surface area for quenching by the polymer.

The slower part of the TRL decay (Fig. 2e) is mediated by exciton diffusion to an interface with the polymer and can give us the size of the pure fullerene spheres if the exciton diffusion coefficient in PC_71_BM is known. Measurement of the exciton diffusion coefficient is described in the next section, and gives a value of 1.6 × 10^−4^ cm^2 ^s^−1^ in PC_71_BM. This enables us to describe the dynamics observed in Fig. 2e based on the information obtained in Fig. 2a–c. The most obvious model is spheres of PC_71_BM surrounded by a matrix of PTB7. We assume that excitons diffuse inside the spheres by a random walk and are instantaneously quenched at the surface with PTB7. The diffusion equation was solved analytically with these boundary conditions following Crank^18^, and the number of fullerene excitons in a sphere is given by:


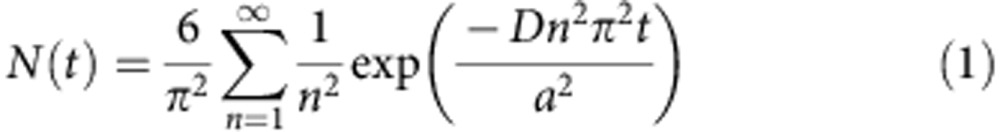


where *a* is the radius of the sphere and *D* the diffusion coefficient which we set to the measured value stated above. Equation 1 was multiplied by the fluorescence intensities in the absence of quencher and convoluted with the instrument response to fit the PL decay.

The simulated decay using a pure 150-nm-diameter sphere of PC_71_BM is displayed in Fig. 2e as the green dashed line and is slower than the measured decay. The best fit with a single sphere model gives a fullerene sphere with a diameter of 60 nm (Fig. 2e, solid blue line), agreeing well with the AFM data (Fig. 2b,c). The good agreement between the directly observed morphology of domains 20–60 nm in diameter with AFM and the deduced fullerene domain size with TRPL indicates that using the decay of PL in combination with the exciton diffusion coefficient in the bulk to model the morphology is valid.

To further test this validity, we turn our attention to the ‘pureness’ of the small spheres of PC_71_BM. We can modify our model by including a small percentage of PTB7 uniformly distributed inside the 60-nm-fullerene spheres (a fuller description is provided in the Methods). We find that even a very small amount (0.2 wt%) of PTB7 mixed into the spheres renders the decay faster than is measured, shown in Fig. 2e as the orange dotted line, giving strong evidence that the small 60 nm spheres are comprised of pure fullerene. To explore this further, we have performed differential scanning calorimetry (DSC) on similar blends of PTB7:PC_71_BM. With DSC, we can monitor the phase transitions of the fullerene in the blend. Such phase transitions (for example, melt temperature) would be modified if significant amounts of the fullerene were in a mixed phase with the polymer, indicating purity. The melt temperature of the fullerene would be the best feature to observe and was found to be at 315–320 °C (see Supplementary Figs S3,S4), consistent with published values^19^,^20^. This fullerene melt cannot be observed in the blend with DSC because of overlapping melt crystallization in PTB7 at this temperature (Supplementary Fig. S3). Information on the fullerene purity can instead be derived from the melt crystallization peak (205 °C)^20^, where there is no overlapping polymer phase transition. Looking at the neat fullerene and the blend, the same melt crystallization peak is observed (see Supplementary Figs S3,S4), thus supporting that the small spheres are composed of pure fullerene.

The overall determined morphology is schematically shown in Fig. 2f, where small pure spheres of fullerene 20–60 nm in diameter in a polymer matrix agglomerate into 150-nm-diameter fullerene-rich domains. These domains sit in a finely mixed phase matrix, and a finely mixed phase skin blankets everything. This morphology is substantially different from those that have been reported using transmission electron microscopy^7^,^10^ and X-ray scattering^8^,^11^. In a report by Chen *et al.*^11^, a hierarchical morphology was observed, with crystallites of a few nanometres forming heterogeneity with a 75-nm-length scale and creating domains of 175 nm size. The work concludes, however, that the domain purity peaks at only ~45%. We are able to determine that the 60-nm-fullerene domains are 100% pure, as indicated in Fig. 2e. In addition, the work by Chen *et al.*^11^ uses PC_61_BM, which has a different miscibility with polymers^21^,^22^,^23^ from PC_71_BM, and thus may produce different morphologies in the blend. The report by Collins *et al.*^8^ suggests pure fullerene domains of 200 nm in size—which they state would limit charge carrier generation. We show that these large domains are not pure and are in fact comprised of smaller pure fullerene spheres 60 nm in size surrounded by polymer. We suggest that the top skin as well as the small-length scales make these structures difficult to detect with previously used techniques.

As noted above, two processes can contribute to quenching of fullerene fluorescence in the blend: charge pair formation and RET to PTB7. Even though RET can occur, the fact that we do not observe PTB7 fluorescence in the blend indicates that fast exciton splitting into charge pairs occurs no matter which process takes place. The efficiency of charge pair formation due to light absorbed on the fullerene can be derived from the fluorescence decay using:


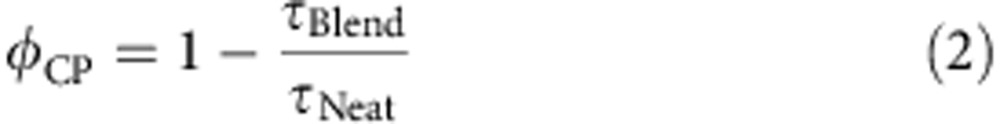


where *τ*_Neat_ is the average fluorescence lifetime of the fullerene in an inert blend with PMMA (600 ps) and *τ*_Blend_ is the average fluorescence lifetime in the photovoltaic blend with PTB7 (67 ps). Hence, for the blend without additive *φ*_CP_=0.1+(0.89 × 0.9)=0.9—that is, 90% of absorbed photons generate charge pairs, where the 0.1 is from directly formed charge pairs in the fullerene as discussed earlier. This number contrasts with an IQE of 40% in the fullerene absorption region, suggesting that only 45% of the initially generated charges are extracted. This is an extraction efficiency under short-circuit conditions and takes into account both geminate and non-geminate recombination losses—that is, all losses that occur between charge generation and charge extraction at short-circuit conditions. Photoconductive–AFM of this blend in Supplementary Fig. S5 shows low photocurrent in the regions between the fullerene-rich domains, which indicate poor electron extraction from polymer-rich domains. This is consistent with an IQE of ~50% in the spectral range of polymer absorption without additive, despite total quenching of the polymer fluorescence. We conclude that even though good exciton dissociation is achieved, large fullerene-rich domains limit charge extraction and OPV performance.

### Exciton diffusion in PC_71_BM

Exciton diffusion in PC_71_BM is important in its own right, as the fullerene is used in many efficient OPV blends with the polymers PCDTBT^24^, PSBDTBT^25^,^26^ as well as PTB7 (refs 7, 16, 27, 28). To determine the exciton diffusion coefficient, we have dispersed electron donor-functionalized molecules into the PC_71_BM film and measured the time-resolved luminescence (TRL) of PC_71_BM to tell us about the degree of PL quenching.

**Figure 3:**
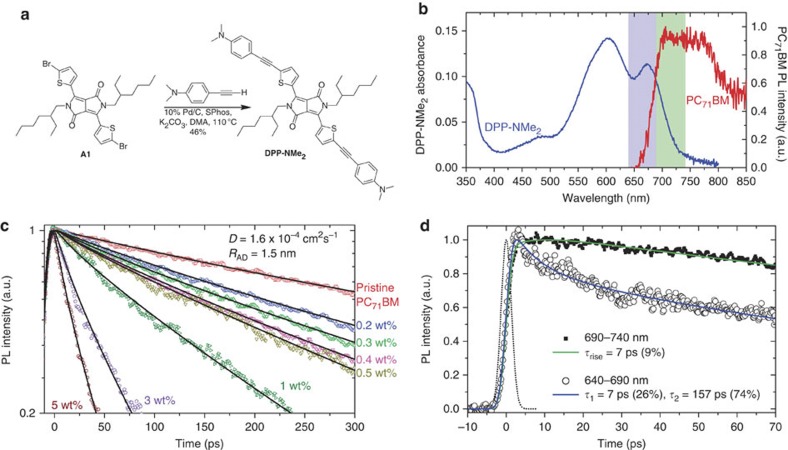
Exciton diffusion in PC_71_BM. (**a**) Chemical synthesis scheme of the electron donor-functionalized diketopyrrolopyrrole derivative DPP-NMe2, with synthesis described in the Methods. (**b**) Absorption spectrum of the DPP-NMe2 molecule that is used in the quenching measurements. The PL spectrum of PC_71_BM is also shown, indicating that there is some spectral overlap between the fullerene emission and DPP-NMe2 absorption. The shaded vertical bands indicate two spectral regions of fullerene emission, 640–690 nm and 690–740 nm—these regions were used to monitor spectral diffusion of the fullerene exciton and thus determine the hopping time. (**c**) PL quenching of PC_71_BM emission when varying the concentration of the DPP-NMe2 quencher from 0 to 5 wt%. The solid lines are fits to the experimental data using the model as defined in the text. The deduced diffusion coefficient as defined in the text is *D*=1.6 × 10^−4^ cm^2^ s^−1^ and radius *R*_AD_=1.5 nm. (**d**) PL dynamics of PC_71_BM when looking at two spectral windows, 640–690 nm (open circles) and 690–740 nm (closed squares), as defined in **b**. A PL decay is observed on the blue side with a best fit (solid line) decay time constant of 7 and 157 ps. In the spectral window 690–740 nm, a rise-time is fitted (solid line) of 7 ps. The instrument response function is shown as a dotted line (3 ps full-width half-maximum).

Small known concentrations of DPP-NMe2 were added to PC_71_BM films. The synthesis of DPP-NMe2 is described in the Methods; synthesis route is shown in Fig. 3a; and absorption spectrum shown in Fig. 3b. Increasing concentrations of the quencher caused a reduction in the excited state lifetimes of the blend relative to the pristine film as measured by TRL. In this low-concentration regime, the molecules of DPP-NME2 are assumed to be randomly distributed throughout the film. In the simplest model, these molecules act as quenching sites so that an exciton in the PC_71_BM will decay if it encounters DPP-NMe2 in the course of its random walk. Mathematically, this can be expressed as the solution to the diffusion equation in spherical coordinates, using the boundary condition that the concentration of the mobile species is held at zero when it gets to within a critical distance, *R*_c_, from a quenching site. This relation defines the rate of quenching of the diffusing species and is known as the Smoluchowski equation^29^. There is spectral overlap between the emission of PC_71_BM and the absorption of DPP-NMe2 (see Fig. 3b), which means that it is possible for some quenching at a distance away from the quencher via resonance energy transfer, rendering our simply collisional boundary conditions unphysical. It was found, however, that the very low PLQY of PC_71_BM means that the Förster radius is relatively short and is hence in the regime that has been calculated by Klein *et al.*^30^ to be well approximated by the Smoluchowski equation. Thus, the rate of change of population of excitons can be described according to equation 3:


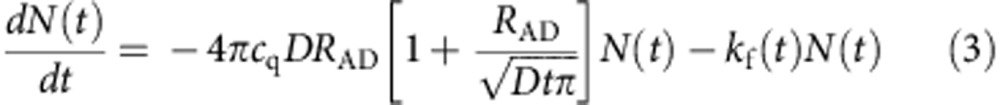


where *N*(*t*) is the population of excitons, *c*_q_ is the concentration of quenching sites in the films, *D* is the exciton diffusion coefficient, *R*_AD_ is a distance of closest approach, inside which the exciton is quenched more rapidly than can be resolved via picosecond TRL measurements. *k*_f_(*t*) is the rate at which the excitons undergo fluorescence decay and was calculated from a 2-exponential fit to the PL emission from the pristine PC_71_BM film. Equation 3 was solved analytically, convoluted with the instrument response and then fitted to the time-resolved fluorescence decays of films with seven different low concentrations of the DPP-NMe2 quencher as shown in Fig. 3c, using *D* and *R*_AD_ as the only fitting parameters. This generated a value of 1.5 nm for the distance of closest approach and an exciton diffusion coefficient in PC_71_BM of 1.6 × 10^−4^ cm^2 ^s^−1^.

An upper limit on the exciton diffusion coefficient can also be estimated from the characteristic time the exciton takes to undergo the first hopping step in its diffusion. The initial hop can be observed in excitons that move from a higher-energy chromophore to a lower one because the energy migration is exhibited in a spectral shift in the fluorescence to longer wavelength at longer time. Figure 3d shows the time-resolved PL in the spectral region 640–690 nm—defined as the blue side of the steady-state PC_71_BM PL spectrum, along with dynamics in the spectral region 690–740 nm, around the steady-state PL peak—both wavelength regions are indicated in Fig. 3b. On the blue side, a fast decay with a time constant of 7 ps is observed, while on the PL peak no decay is recorded, and instead a small rise-time of 7 ps is required for a good fit. The differences in dynamics at these two wavelengths indicate that the fast decay is not related to a loss of oscillator strength or electronic reconfiguration, but rather is consistent with spectral relaxation because of exciton hopping. Consequently, a time for the first hop of 7 ps can be deduced.

Exciton diffusion is expected to slow down with time because excitons relax to low energy sites and the 7-ps-hopping time can be used to determine the diffusion coefficient’s initial value. By making the simplifying approximation that the PC_71_BM molecules lie on a cubic lattice, the diffusion coefficient can be determined using^29^:


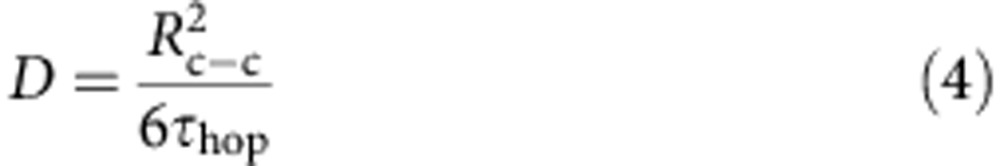


where *R*_c-c_ is the centre to centre distance and *τ*_hop_ is the characteristic hopping time. Using a density of a similar material, PC_61_BM, reported^31^ to be 1.3 g cm^−3^, the distance between chromophores on a cubic lattice is calculated as 1.3 nm. This gives the initial diffusion coefficient of 3.6 × 10^−4^ cm^2 ^s^−1^. This is twice higher than the effective long-time value determined by the volume quenching technique. A similar value has been reported^32^ of *D*=2 × 10^−4^ cm^2 ^s^−1^ for the other fullerene derivative, PC_61_BM.

The one-dimension (1-D) diffusion length (*L*_*D*_) can be calculated using the relation:


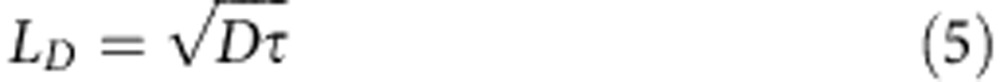


Using the values of *D* and *τ* measured, *L_D_*=3.1 nm.

### Blends prepared with additive

Solvent additives have been shown to greatly enhance device performance in a number of OPV blends^7^,^8^,^33^. Past studies have also shown that DIO increases intermixing of fullerene derivatives with PTB7 and suggested that proximity of the polymer to pure fullerene aggregates are important for efficient charge separation and transport^10^,^11^. Here we show that DIO dramatically improves the extraction of photogenerated charges, which can be explained by the formation of narrow elongated fibre-shaped fullerene-rich and polymer-rich domains, with a continuous change in composition observed by photocurrent mapping implying a concentration gradient between the two materials.

**Figure 4:**
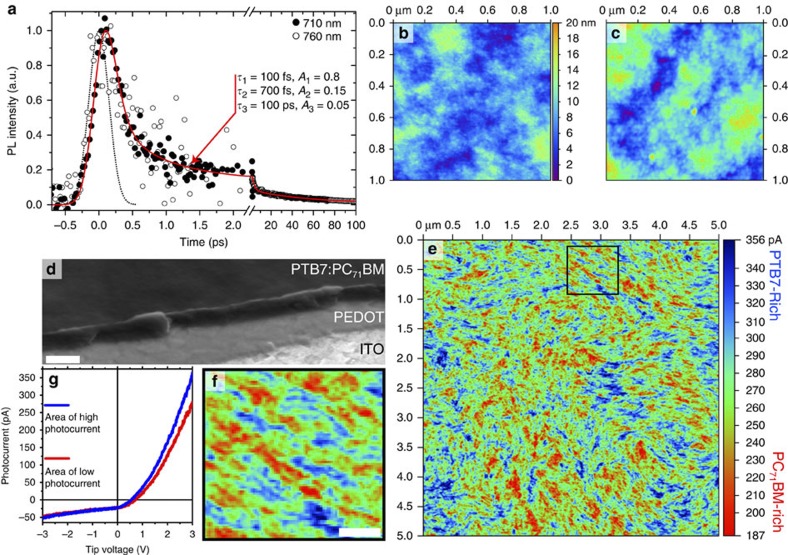
Results of PTB7:PC_71_BM blends prepared with 3% DIO. (**a**) Ultrafast fluorescence dynamics recorded at 710 nm (closed circles) and 760 nm (open circles), representing fullerene emission. The best fit to the data (red line) gives decay time constants of 100 fs representing 80% of the amplitude and 700 fs representing 15%. A significantly slower 100-ps decay is observed to make up the remaining 5% of amplitude and is measured with a streak camera. The instrument response function for the decay up to 2 ps is shown as a dotted line and is 350-fs full-width half-maximum. Shown in **b** is the topography measured with AFM of the sample as made and in (**c**) after removing ~20 nm of material with plasma-ashing. The *z*-scale shown between the two panels is common to both topographies. In **d** an SEM image of the blend is shown, viewed from an angle of 52° with a score made across the film, enabling the ITO, PEDOT and blend to be imaged in profile, where the white scale bar is 500 nm. (**e**) Photoconductive–AFM image of a 5 × 5 μm window under 670 nm excitation with +3 V tip bias, the *z*-scale is photocurrent in units of picoamps. High photocurrent (blue) indicates regions that are polymer-rich, with low photocurrent (red) indicating fullerene-rich regions. Shown in **f** is a close-up of the black square from **e**, looking at the morphology of the two materials in detail, the white scale bar is 200 nm. (**g**) Photocurrent–voltage characteristics for a typical region of high (blue line) and low (red line) photocurrent.

First, we can obtain information on the degree of mixing between the two materials using TRL. The PL decay of the blend prepared with DIO at 710 nm is shown in Fig. 4a as filled circles and it displays very fast quenching of fullerene fluorescence by the polymer. The fit to a three-exponential function gives a decay time constant of 100 fs for 0.8 fraction of the fullerene, a slower 700 fs component (of fraction 0.15) and a slow component of 100 ps for 0.05 fraction—mis-fits showing the poor fitting that is achieved when any of these values deviate significantly from our best fit are shown in Supplementary Figs S6–S8. While the exponential fitting of the PL decay has no absolute physical meaning, it can tell us directly about the timescale of the dissociation of excitons into charge pairs in the blend, where the very fast decay of exciton emission agrees with the reported charge formation on a sub-picosecond timescale^34^. This qualitatively suggests that the fullerene molecules are in close proximity to the polymer and that the fullerene excitons do not need to transverse regions of fullerene via exciton diffusion to reach the PTB7, in contrast to the blend without DIO where more fluorescence persists for longer as the fullerene excitons diffuse to the polymer. PTB7 fluorescence in neat films has a peak at 810 nm; therefore, it is possible to distinguish between fullerene and polymer fluorescence spectrally, especially because the PTB7 radiative rate is almost twenty times that of the fullerene (0.44 versus 0.025 ns^−1^, as described in the Methods). Shown in Fig. 4a is ultrafast emission at 760 nm (open circles), where the PTB7 fluorescence intensity is ~0.65 of its peak intensity, and the dynamics are identical to emission at 710 nm. This indicates that all the emission we detect is from the fullerene and no polymer emission is detected—that is, the PTB7 excitons are dissociated extraordinarily quickly (<100 fs)—and suggests that polymer chains are intimately mixed with fullerene, agreeing with previous morphological studies^7^,^8^,^9^.

The charge pair generation efficiency from absorbed photons in the blend with DIO is calculated using equation 2, and experimental values for *τ*_Neat_=220 ps and *τ*_Blend_=5.2 ps give a charge pair formation efficiency, *φ*_CP_=0.98. The IQE in devices prepared with DIO is 80% in the fullerene absorption region and 90% in the polymer absorption region. Since the charge generation is nearly unity, IQE can be taken as a measure of the charge extraction efficiency at short-circuit conditions, including all losses after charge generation in both cases. Intimate mixing will give large interfacial areas for efficient charge formation; however, charge pairs formed at the donor–acceptor interface are Coulombically bound and will recombine geminately^35^,^36^,^37^,^38^,^39^ unless they are extracted by continuous networks of polymer and fullerene. Consequently, we must look at the morphology to explain the efficient charge extraction.

Measuring the surface topography with AFM of the as spun film (Fig. 4b) and after plasma-ashing that removes ~20 nm of the material (Fig. 4c) shows similar topographies, while a profile of the blend with SEM (Fig. 4d and Supplementary Figs S9–S12) shows no obvious lateral structure, indicating that morphologies of the surface and of the bulk film are not substantially different. This contrasts with morphology in the blend without DIO. Recently it has been shown^40^,^41^,^42^,^43^,^44^ that photocurrent mapping with photoconductive–AFM (pc-AFM) gives high resolution and high-contrast morphological information and we have therefore used this technique to study blend morphology. A pc-AFM image of the PTB7:PC_71_BM blend prepared with DIO and acquired with a tip voltage of +3 V and 670 nm red light excitation is shown in Fig. 4e and a zoomed area in Fig. 4f. Dark current in the same area was at least five times lower than with 670 nm light. Details of the pc-AFM experiments and photocurrent maps with negative tip bias as well as maps at different positive tip bias are given in the Methods and Supplementary Figs S13,S14. Figure 4g shows typical photocurrent–voltage curves of high and low current regions. Without an applied bias, photogenerated electrons cannot flow to the gold tip owing to a Schottky barrier but they can be extracted to the indium tin oxide (ITO), while holes can be extracted to the gold tip, generating a negative photocurrent. With a positive tip bias higher than the open circuit voltage (*V*_OC_) of ~0.7 V, electrons overcome the Schottky barrier and are extracted to the tip, whereas holes flow to the ITO and the photocurrent is positive. The absolute value of the negative photocurrent is about 5–10 times lower for the same voltage, which can be explained by a barrier to extract photogenerated holes from the tail of the density of states of the polymer into the gold tip. The 670-nm light preferentially excites the polymer; therefore, high photocurrent is likely to correspond to polymer-rich regions. This is confirmed by the observation that photocurrent maps obtained at negative tip bias correlate with positive tip bias maps (see Supplementary Fig. S13) as well as confirming that the measured quantity is photocurrent. Consequently, lower photocurrent is from fullerene-rich regions of the sample. The first thing to notice in the photocurrent map is that the domains of high and low photocurrent are smaller and more elongated than those without the additive, which indicates that polymer-rich and fullerene-rich domains have a high aspect ratio, with their lengths being longer than their widths. The regions of the blend with the highest and lowest photocurrents are narrow and resemble elongated thin fibre-like strands or swirling ‘brushed fibres’, with the close-up image shown in Fig. 4f indicating that these richest domains are typically 10–50 nm wide and 200–400 nm long. The photocurrent values show a gradual change going from high to low current regions, as indicated in Supplementary Fig. S15. The same regions of high photocurrent are observed in the range of +1.5 to +6 V (see Supplementary Fig. S14), which indicates that photocurrent contrast is not affected by charge extraction; thus, the photocurrent contrast is dependent on the polymer concentration as it is absorbing the 670-nm tip light. Therefore, a continuous change of photocurrent in the pc-AFM map implies that the mixing ratio of the polymer and fullerene also changes gradually. The interdiffusion of the polymer and fullerene to form mixed phases has been observed in a number of OPV blends^45^,^46^,^47^,^48^,^49^ and can have an important role in device performance.

Previous studies have not reported any sort of fibre-like morphology in these systems, instead observing hierarchical morphology^11^ little different than without additive when PTB7 is blended with PC_61_BM. When PC_71_BM was blended with PTB7, Collins *et al.*^8^ were able to identify that on a length scale larger than ~100 nm that the fullerene purity varied from 52 to 68%, while the average fullerene domain size was measured to be a distribution centred around 30 nm, ranging from 6 to 200 nm. This domain size was unable to determine any spatial organization of the domains, and so our work observing the elongated fibre-like alternating phases of polymer-rich and fullerene-rich domains enables the lengths observed by Collins *et al.*^8^ to be placed in a spatial context.

It should be noted that while the pc-AFM image shows the morphology in-plane, and that in a working device charge transport will be perpendicular to the plane (towards the contacts), our understanding is that the concentration gradient of each material that forms between the polymer-rich and fullerene-rich regions helps the dissociation of geminate charge pairs by diffusion as positive/negative charge diffuses preferentially towards the polymer-rich and fullerene-rich domains, respectively. In pc-AFM, the charges are extracted by a strong electric field, whereas in working OPVs the built-in field is much weaker and charge diffusion has an important role in separation of Coulombically bound charge pairs^39^. Directional charge diffusion driven by a concentration gradient is expected to lead to more dissociation than a random walk. The separated charges are then transported in the fibre-like polymer-rich and fullerene-rich domains with low probability of recombination. The overall morphology is thus beneficial for exciton dissociation into a charge pair, geminate pair dissociation into free charges and charge extraction, all of which are consistent with the observed high IQE of 80–90%. Rather than simply being an agent to ensure that the materials are well mixed, the solvent additive creates an environment where the optimum morphology for a working device is created.

## Discussion

The results presented have enabled significant new nanoscale morphology of the high-performance PTB7:PC_71_BM blends to be observed for the first time, determining how the morphology of the blend affects the efficiency of charge carrier generation, extraction and the quantum efficiency of OPV devices. We show that blends prepared from solutions without any processing additive form large (100–200 nm) fullerene-rich domains comprised of small 20–60 nm pure fullerene spheres. The exciton dissociation efficiency is high (90%) in these blends but the IQE is only 40–60%, limited by charge extraction. This indicates that large fullerene- and polymer-rich domains give poor charge extraction and high recombination, leading to low quantum efficiencies. By using the additive DIO, narrow and extended fibre-like polymer-rich and fullerene-rich domains are created, with fibres 10’s of nanometres wide and 100’s of nanometres long for both materials. Exciton dissociation in the blends prepared with DIO is very fast, with 95% of exciton decay amplitude occurring on a sub-picosecond timescale. The fibre-like polymer-rich and fullerene-rich domains form part of a continual change in the polymer-fullerene mixing ratio when going from one to another, implying a concentration gradient that is favourable for charge pair dissociation and minimizes geminate recombination, thus giving high extraction efficiency (80%) of photogenerated charges. Consequently, our study of the PTB7:PC_71_BM system tells us about the optimum morphologies that are required, and in this system created, for efficient OPV devices.

Although our study has looked specifically at PTB7:PC_71_BM blends, parallels and contrasts in the morphology can be drawn with other high-performance OPV blends. Charge transport modelling indicates the importance of continuous phases of the donor and acceptor materials and good connectivity to the appropriate electrodes, whereas the domain size has been found to be less important^50^. The extended fullerene-rich and polymer-rich domains observed in our work give efficient charge extraction, indicating that they are well connected, despite being only 10–20 nm wide. We note that fibrous morphology has been observed in P3HT- (ref. 51), PCDTBT- (ref. 24) and PCPDTBT (ref. 52)-based blends with PCBM. However, fibres were observed only in the polymer phase. We see that not only the polymer but also the fullerene show this kind of spatial organization, giving bi-continuous networks for both electron and hole transport. Short-range ordering of PTB7 has been explored with grazing-incidence X-ray diffraction^10^ and indicates that in the blend ~20% of the polymer can be considered crystalline. We are therefore left to consider that bundles of extended PTB7 chains form a template that helps self-organization of fullerene molecules around them, forming fibrous strands of both the donor and acceptor materials, which are advantageous for efficient OPV performance. Owing to the spatial resolution of pc-AFM, it is also possible that both polymer-rich and fullerene-rich domains contain regions of a few nanometres in width of pure material, which are too small for us to observe. The optimum morphology in PTB7:PC_71_BM is substantially different from previous generations of BHJ blends and suggests that the properties of PTB7 are different from other conjugated polymers used in OPVs and can be exploited in future material development.

## Methods

### Sample preparation

PTB7 was purchased from 1-Material and had a molecular weight of 92,000 Da with a polydispersity of 2.6. PC_71_BM of 99% purity was purchased from Solenne. 1,8-DIO was purchased from Fluka and chlorobenzene (HPLC grade) from Sigma Aldrich. Samples of the blend were prepared with parameters as described in Liang *et al.*^7^ and He *et al.*^16^ In short, 10 mg of PTB7 and 15 mg PC_71_BM were dissolved in 1 ml of chlorobenzene at 50 °C with gentle stirring in a nitrogen-filled glovebox. After letting the solution cool, blends without additive were then spun directly on to clean fused silica disks at 1,000 r.p.m. in the glovebox. For the samples with DIO, 3% by volume of DIO was added to the solution and left to stir for 5 min prior to spin-coating with the same parameters.

### Time-resolved PL

Measurements were performed on two setups; femtosecond data were acquired with upconversion spectroscopy (FOG100 from CDP systems), while picoseconds–nanosecond data were acquired with a synchroscan streak camera (C6860 from Hamamatsu). The excitation in both cases was the frequency doubled output of a Ti:sapphire laser, giving 400 nm 100 fs full-width half-maximum pulses at 80 MHz. Samples for streak camera measurements were held under an active vacuum of ~10^−5^ mbar. Samples for upconversion measurements were encapsulated by sealing with a fused silica window in a nitrogen-filled glovebox and were rotated during measurements, with no degradation visible over the length of the scan.

### Device fabrication

ITO-coated glass substrates (15 Ω per square) from Xin Yan Technology Ltd were masked and etched in hydrochloric acid (37%) for 20 min. The mask was removed and the substrates cleaned by sonication in deionized water, acetone and isopropanol. The substrates were then dried with nitrogen and treated in an oxygen plasma asher for 5 min. Poly(3, 4-ethylenedioxythiophene:poly(styrenesulfonate) (PEDOT:PSS)(Clevios AI4083) was spin-coated at 4,000 r.p.m. The PEDOT:PSS-coated ITO substrates were annealed on a hotplate at 120 °C for 20 min before being placed in a nitrogen-filled glovebox and PTB7:PC_71_BM spin-coated on top with the same conditions as noted above. The substrates were then inserted into an evaporator for top electrode deposition. An ~20-nm calcium layer and an ~200-nm aluminium cathode were thermally evaporated at a pressure of 2 × 10^−6^ mb. Immediately after top electrode deposition, the devices were removed from the evaporator and encapsulated with a UV optical adhesive and a glass coverslip. Devices were then removed from the glovebox, masked and characterized under an illumination intensity of 100 mW cm^−2^ in air using a K.H. Steuernagel AM1.5G solar simulator and a Keithley 2400 source-measure unit. The illumination intensity was verified with an NREL-calibrated monosilicon detector and a KG-5 filter. EQE measurements were obtained with an incident photon to charge carrier efficiency setup, which consists of an NPL-calibrated photodiode, Keithley 6517A picoammeter and a TMc300 monochromator.

### SEM and AFM

The dual beam system Nova Nanolab (FEI Co.) was employed for SEM imaging. The sample was tilted by 52 degrees and the cross-section of device was imaged near the scratch. AFM measurements were obtained with an SPM Solver Next (NT-MDT) AFM. Topography and phase images were measured in tapping mode with repulsive average tip-sample force by using probes NSG11 (NT-MDT).

### Photoconductive–AFM

Pc-AFM was performed with an SPM Solver P47H (NT-MDT) in a nitrogen-filled glovebox. Conductive gold-coated probes NSC36/Cr-Au and CSC37/Cr-Au (Micromash) with three levers on each probe were used for pc-AFM measurements. All measurements were performed with the longest lever on chip. Photoexcitation to enable photoconductivity measurements was provided by the AFM laser at a wavelength of 670 nm. We confirmed that photocurrent was measured by scanning a region with the 670-nm tip light on to determine the topography and conductivity, and then rescanned the same area with the 670-nm tip light off and measuring only conductivity, using the tip-sample distance derived from the first scan—in this scenario we see current with the tip light on but none on the second pass with the tip light off, as shown in Supplementary Fig. S5 along with a description of the geometry of the AFM tip laser, enabling direct illumination of tip-sample contact area. The sample was grounded and the denoted voltage was applied to the tip. The sample comprised of an ITO-coated glass substrate on which PEDOT:PSS and then PTB7:PC_71_BM were deposited by spin-coating with the same conditions as noted above.

### Optical modelling

The optical density and absorption simulations were performed using finite-element in the COMSOL package. The refractive indexes of the PTB7:PC_71_BM blend and of PEDOT used in the simulations were measured by ellipsometry as shown in Supplementary Fig. S16. The refractive indexes of ITO, Calcium and Aluminium were taken from the literature. By deriving the true active layer absorption with optical modelling, all interference effects in the stack are taken into account, and thus the IQE spectra can be deduced directly from the EQE spectra.

### Synthesis and characterization of DPP-NMe_2_

Compound **A1**^53^ (600 mg, 0.88 mmol), as shown in Fig. 3a, 10% Pd/C (103 mg, 0.10 mmol), SPhos (40 mg, 0.10 mmol) and K_2_CO_3_ (305 mg, 2.20 mmol) were dissolved/suspended in dimethylacetamide (5 ml). The mixture was degassed with N_2_ for 5 min, and then 4-ethynyl-*N,N*-dimethylaniline (282 mg, 1.94 mmol) was added to the mixture. The solution was heated at 110 °C overnight. The reaction was cooled to room temperature, diluted with ethyl acetate and washed repeatedly with brine. The organic layer was separated, dried over MgSO_4_, filtered and the solvent removed under reduced pressure to afford a crude product that was subjected to silica gel column chromatography (petroleum ether/dichloromethane (1:1)) to afford the product as a blue metallic solid (0.32 g, 45%). Mp. 275 °C (Dec.). ^1^H NMR (400 MHz, CDCl_3_) *δ*=0.90 (12H, m), 1.22–1.42 (16H, m), 1.90 (2H, m), 3.02 (12H, s), 4.01 (4H, m), 6.66 (4H, *J=*9.0 Hz, d), 7.30 (2H, *J=*4.2 Hz, d), 7.41 (4H, *J=*9.0 Hz, d), 8.92 (2H, *J=*4.2 Hz, d). ^13^C NMR (100 MHz, CDCl_3_) δ=10.5, 14.0, 23.0, 23.6, 28.3, 30.1, 46.1, 46.0, 40.2, 80.8, 99.8, 108.5, 108.7, 111.8, 129.5, 130.0, 131.8, 132.8, 135.8, 139.4, 150.6, 161.7. MS (FAB/NOBA): m/z 810.5 [M+H]+. Anal. Calcd for C_50_H_58_N_4_O_2_S_2_: C, 74.03; H, 7.21; N, 6.91. Found: C, 74.17; H, 7.42; N, 6.88.

### Pureness of fullerene spheres

The ‘pureness’ of the small spheres of PC_71_BM can also be determined using the time-resolved PL data. This can be modelled by using the product of the normalized populations of the solution to the differential equation (equation 3) and the population in the sphere quenching case (equation 1). The polymer was assumed to be outstretched and an effective radius was calculated from the length of the chain (~140 nm for 92,000 Da MW, assuming equilibrium bond lengths) and a capture radius from the chain approximated as 1 nm (reasonable in comparison to the capture radius calculated from the DPP-NMe2 complex). These parameters were used to approximate the straight polymer as a highly prolate spheroid, which can be used to generate an effective spheroidal capture distance of 14 nm using the method described by Sreearunothai *et al.*^54^ The simulated decay for a 60-nm-diameter sphere of PC_71_BM with 0.2 wt% of PTB7 homogenously mixed in to it is faster than is observed experimentally (Fig. 2e), suggesting that even a very small amount of PTB7 in the sphere causes significant quenching. Consequently, we can conclude that the spheres have to be pure fullerene, as even a very small amount of PTB7 causes more quenching than is observed.

### Determination of PC_71_BM to PTB7 Förster radius

To determine the Förster radius, *R*_0_, for RET from the PC_71_BM donor to the PTB7 acceptor we use equation 6 (ref. 55):


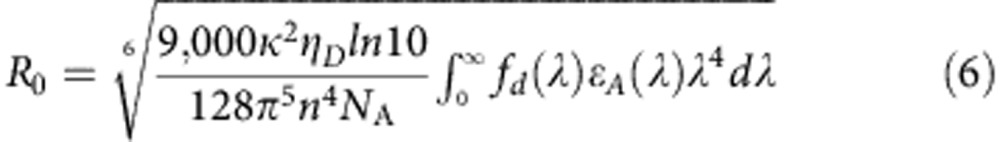


where *κ* is the orientation factor between the donor and acceptor and is taken to be 2/3 for randomly orientated dipoles, *η*_*D*_ is the PL quantum yield of the donor (measured to be 0.015 for PC_71_BM doped in PMMA at 1% concentration), *n* is the refractive index of the film (taken to be 1.9), *N*_A_ is Avogadro’s number, *f*_*d*_(λ) is the PC_71_BM fluorescence spectrum normalized to area 1 and *ε*_*A*_(λ) is the molar absorption coefficient spectrum of PTB7, with values derived from a solution of PTB7.

With the values defined above, and the spectral overlap as shown in Fig. 1b we calculate that the Förster radius is 2.17 nm.

### Determination of radiative rates for PTB7 and PC_71_BM

To determine the radiative rate, *k*_R_, of the PL for PTB7 and PC_71_BM we use equation 7:


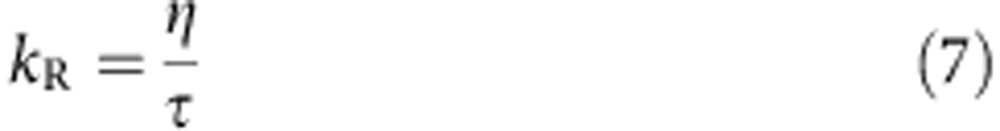


where *η* is the PLQY and *τ* is the natural lifetime. For PTB7, *η*=0.02 and *τ*_ave_=45 ps, thus *k*_R_=0.44 ns^−1^. For PC_71_BM, *η*=0.015 and *τ*_ave_=600 ps, giving *k*_R_=0.025 ns^−1^.

### Experimental methodology for DSC

DSC was performed with a Netzsch DSC204F1, using hermetically sealed aluminium pans, with an empty pan used as a reference. The scan of PC_71_BM shown in Supplementary Fig. S4 was measured with a Netzsch STA449 with open air pans. All DSC scans were performed with a scan rate of 10 K min^−1^ with a first heat to 170 °C, then a cool to 30 °C, followed by a second heat to 380 °C. Blend samples were made by solution casting from a solution of PTB7:PC_71_BM made with the same concentration and ratio as used for the spin-coated samples (10:15 mg ml^−1^ dissolved in 1 ml of chlorobenzene at 50 °C with gentle stirring in a nitrogen-filled glovebox). The solution was deposited on a glass microscope slide and left to dry in a nitrogen-filled glovebox for ~3 days to allow the solvent to evaporate. Complete removal of the solvent was assured by cycling the sample under vacuum of ~10^−2^ mbar. The film thickness was determined with a Veeco Dektak 150 surface profiler, making a scratch on the film and measuring the depth. A film thickness of ~4 μm was measured. The film was then scraped off the glass substrate and deposited into the aluminium pan for the DSC measurements.

## Author contributions

G.J.H. and I.D.W.S. planned the research. G.J.H. performed measurements of time-resolved fluorescence on the blends and spectral diffusion in PC_71_BM, made the blend samples for morphology measurements and performed the analysis of pc-AFM and TRPL data with A.R. A.J.W. measured the fluorescence quenching of PC_71_BM by dispersed quencher and carried out the calculations for determining the exciton diffusion coefficient in the fullerene and diffusion-defined morphology in the blends. A.A. measured the AFM, pc-AFM and SEM of the blends. C.T.H. fabricated and characterized the solar cells. E.R.M. measured the optical constants of the materials with ellipsometry and performed the optical modelling. L.A.S. and G.C. synthesized DPP-NMe2 used in the volume quenching experiments. A.R. and I.D.W.S. guided the research. The article was written by G.J.H., A.R. and A.J.W.

## Additional information

**How to cite this article:** Hedley, G. J. *et al.* Determining the optimum morphology in high-performance polymer-fullerene organic photovoltaic cells. *Nat. Commun.* 4:2867 doi: 10.1038/ncomms3867 (2013).

## Supplementary Material

Supplementary Information Supplementary Figures S1-S16
