# Supplementary material for: Determining the optimum morphology in high-performance polymer-fullerene organic photovoltaic cells
Source: Nat Commun. 2013 Dec 17;4:2867. doi: 10.1038/ncomms3867 (PMC3905772; doi:10.1038/ncomms3867)
Supplement: Supplementary Information — Supplementary Figures S1-S16 [file ncomms3867-s1.pdf]

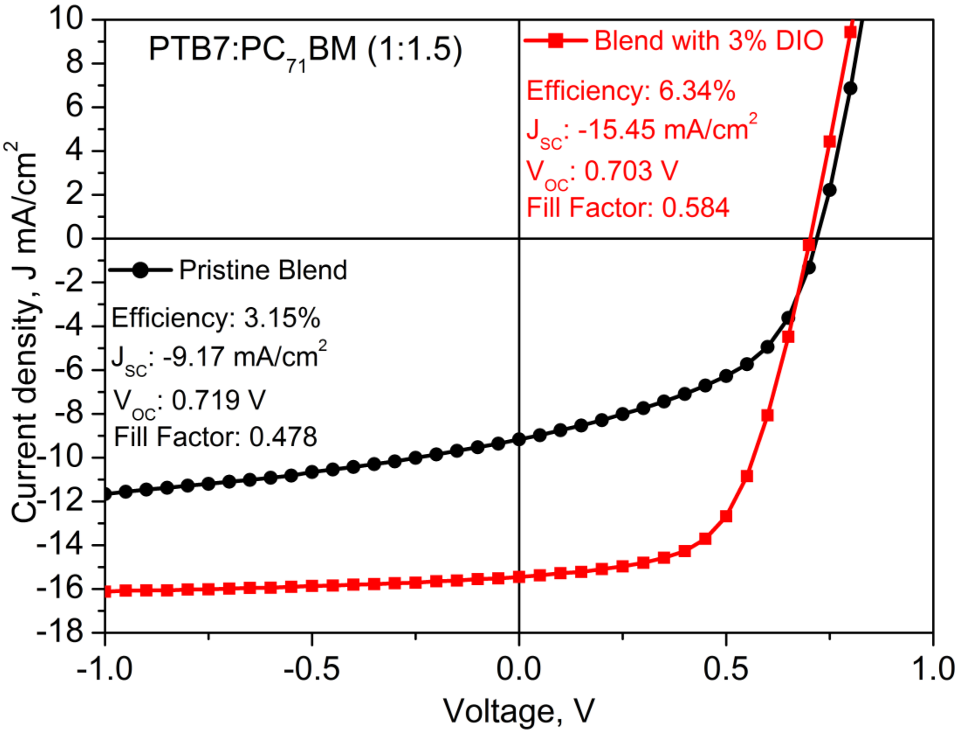

Supplementary Figure S1: PTB7:PC_{71}BM Device Characteristics: Current-voltage dependence of PTB7:PC_{71}BM devices from solutions without additive (solid circles) and from solutions with 3% DIO (solid squares). Device performance parameters are shown in the inset. External and internal quantum efficiency spectra of these same devices are shown in Figure 1 of the main text.

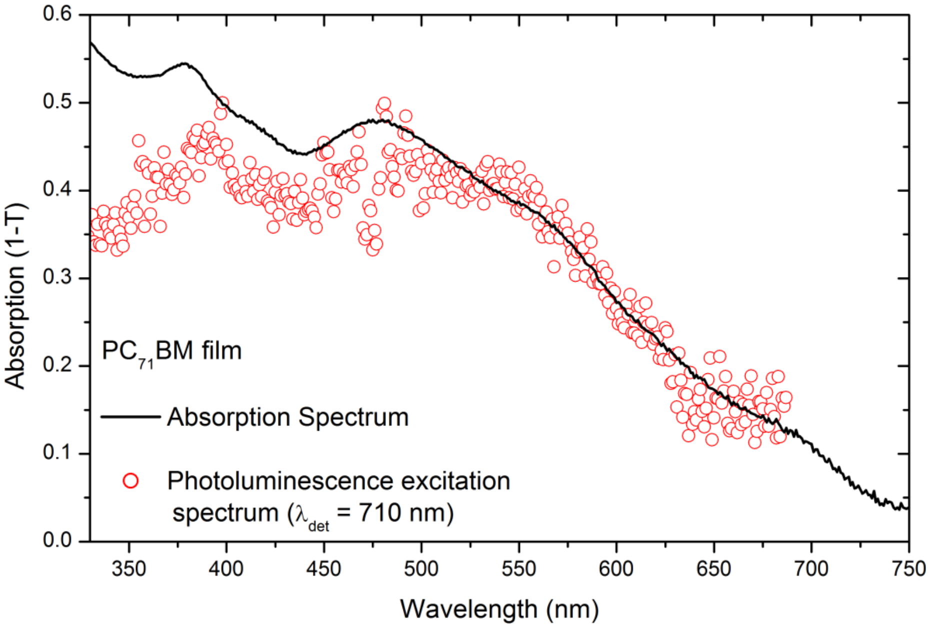

Supplementary Figure S2: Photoluminescence Excitation Spectrum of PC$_{71}$BM: Comparison of steady state absorption spectrum (black line) and photoluminescence excitation spectrum (open red circles) detected at 710 nm of a neat film of PC$_{71}$BM.

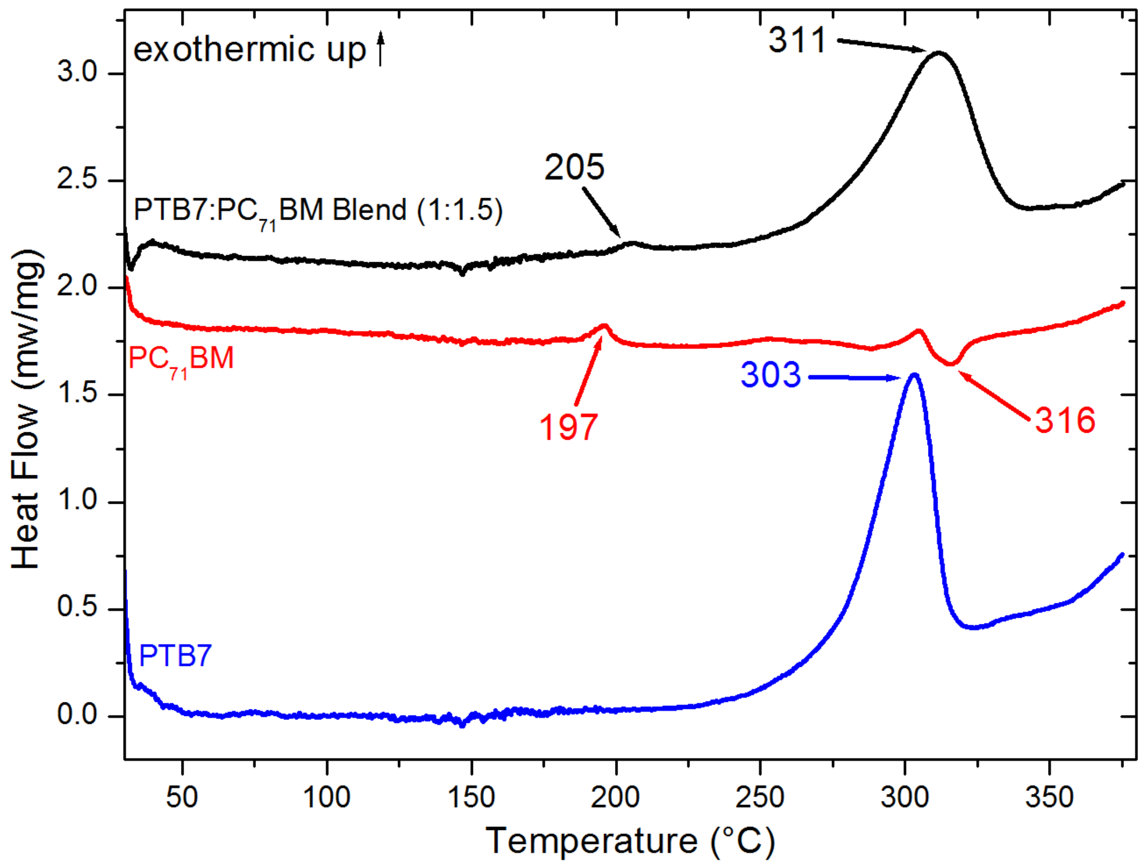

Supplementary Figure S3: Differential Scanning Calorimetry Thermograms: DSC thermograms for the second heating of PTB7 (blue line), $PC_{71}BM$ (red line) and a 1:1.5 ratio blend of the two (black line), with significant peaks annotated. Exothermic heat flow is up on the y-axis.

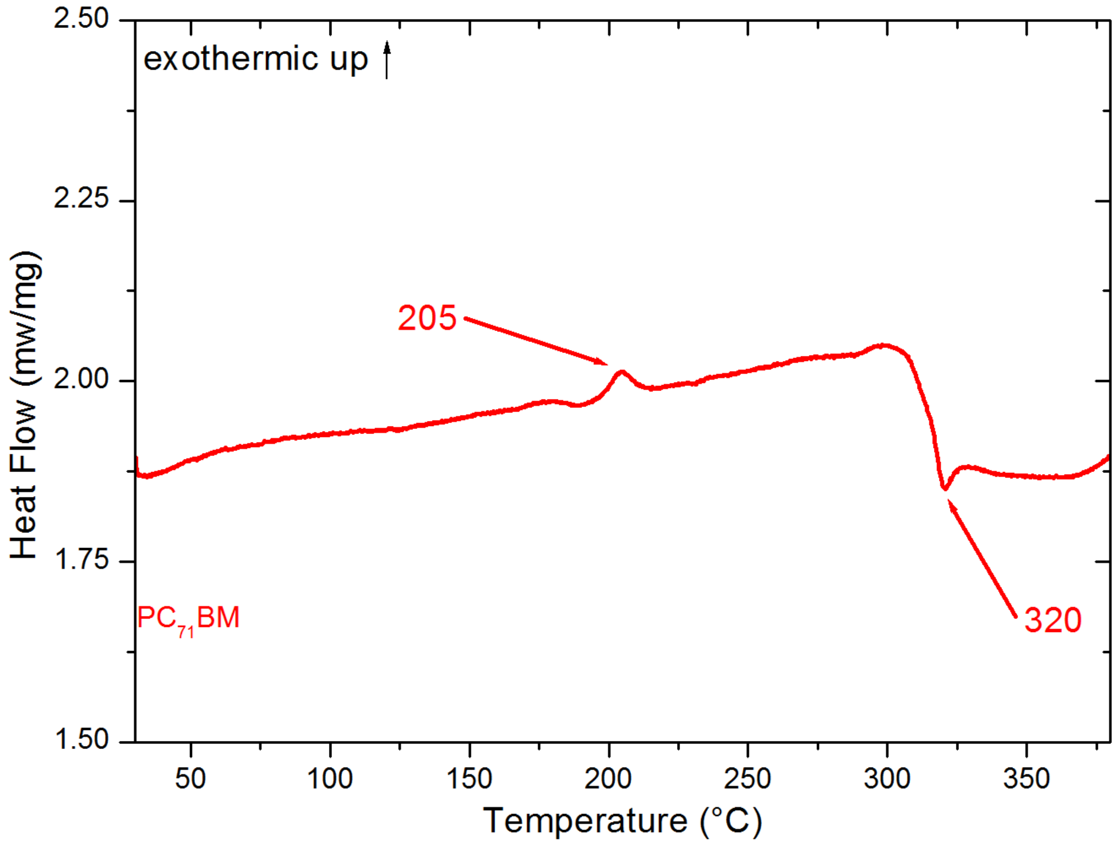

Supplementary Figure S4: Differential Scanning Calorimetry Thermogram of $PC_{71}BM$: DSC thermogram for the second heating of $PC_{71}BM$ (red line) on a Netzch STA449 open air system, with significant peaks annotated.

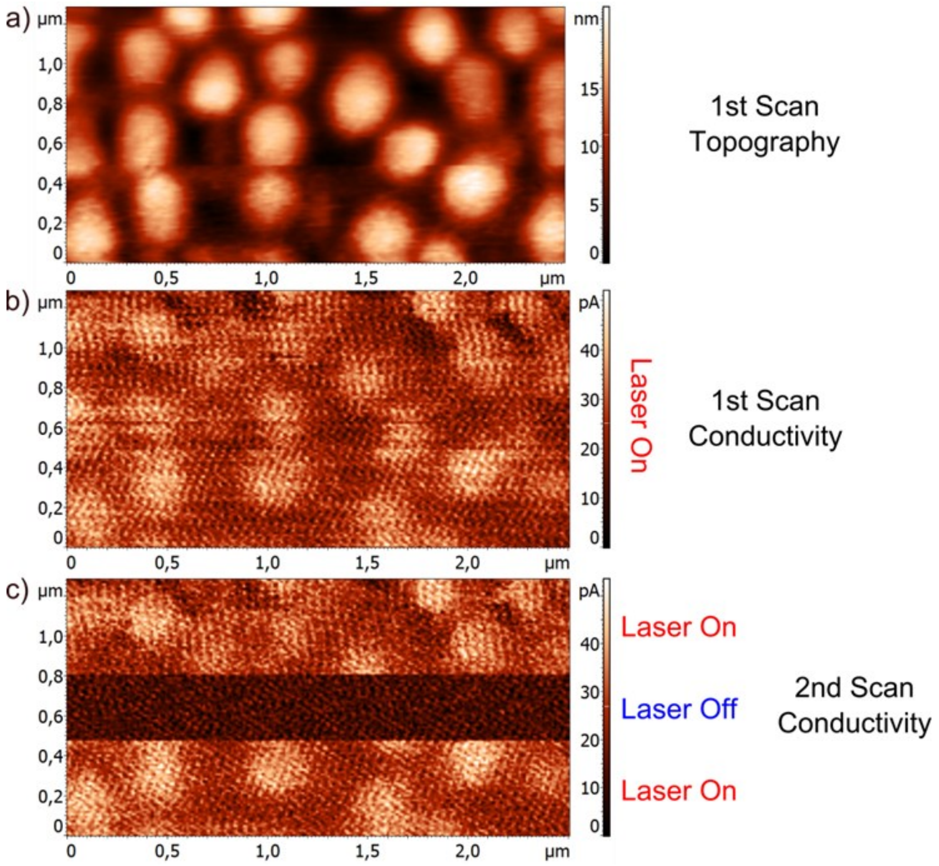

Supplementary Figure S5: Determination That Measured Quantity in pc-AFM is Photocurrent: To test that photocurrent was measured in the pc-AFM experiments we have completed a series of checks. Firstly, the geometry of the AFM lever and tip indicate that the laser illuminates the sample directly. The SPM Solver P47H (NT-MDT) AFM that was used has a laser that illuminates an area wider than the lever at an angle of 45° with respect to the lever. Given the lever-tip dimensions, the 45° excitation angle is enough to enable the laser light to directly illuminate the sample at the point where the point of the tip is passing over it. To directly test the effect of the AFM laser, we completed a scan on an area of a PTB7:$PC_{71}BM$ blend sample without DIO. Shown in **(a)** is the measured the topography and the conductive-AFM with the 670 nm laser light on and a tip bias of +7.2 V **(b)**, both acquired on the first scan. We then made a second conductivity pass of the same region **(c)**, and for the middle part of the scan we switched off the 670 nm laser – using the sample-tip height profile derived from the first scan to determine the tip position. It is clear that measurable conductivity is only produced when the 670 nm laser is on – indicating that we are measuring photocurrent with the setup. Dark current of ~ 20 pA is measured with the 670 nm laser turned off.

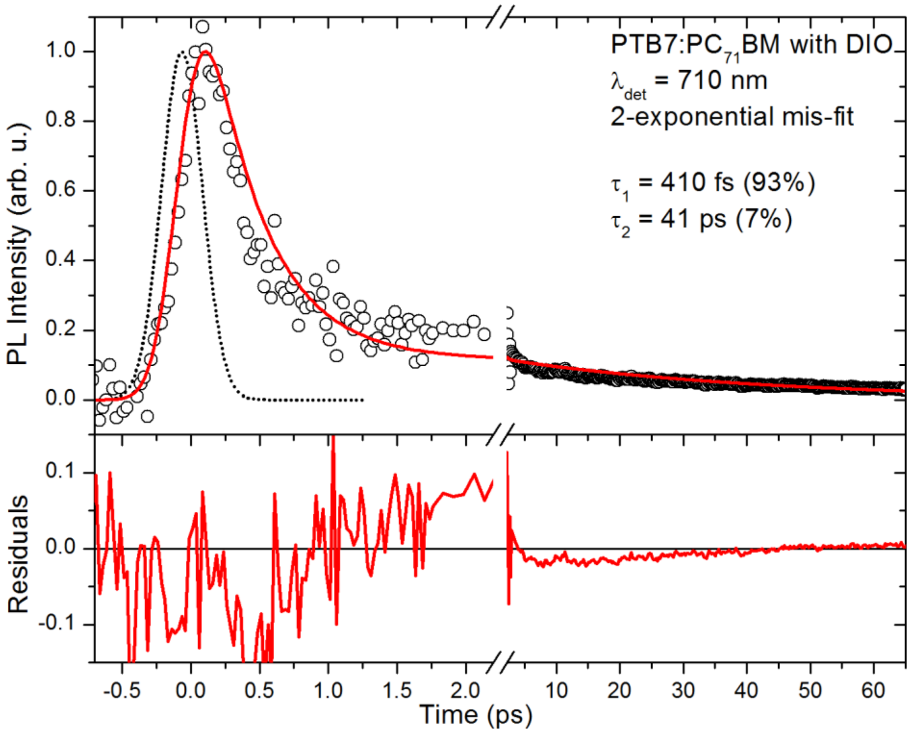

Supplementary Figure S6: Time-Resolved PL Mis-Fit: Only Two Exponentials: PTB7:PC_{71}BM blend with DIO fluorescence decay mis-fit when only two exponential components are used to describe the decay, indicating poorness of fit.

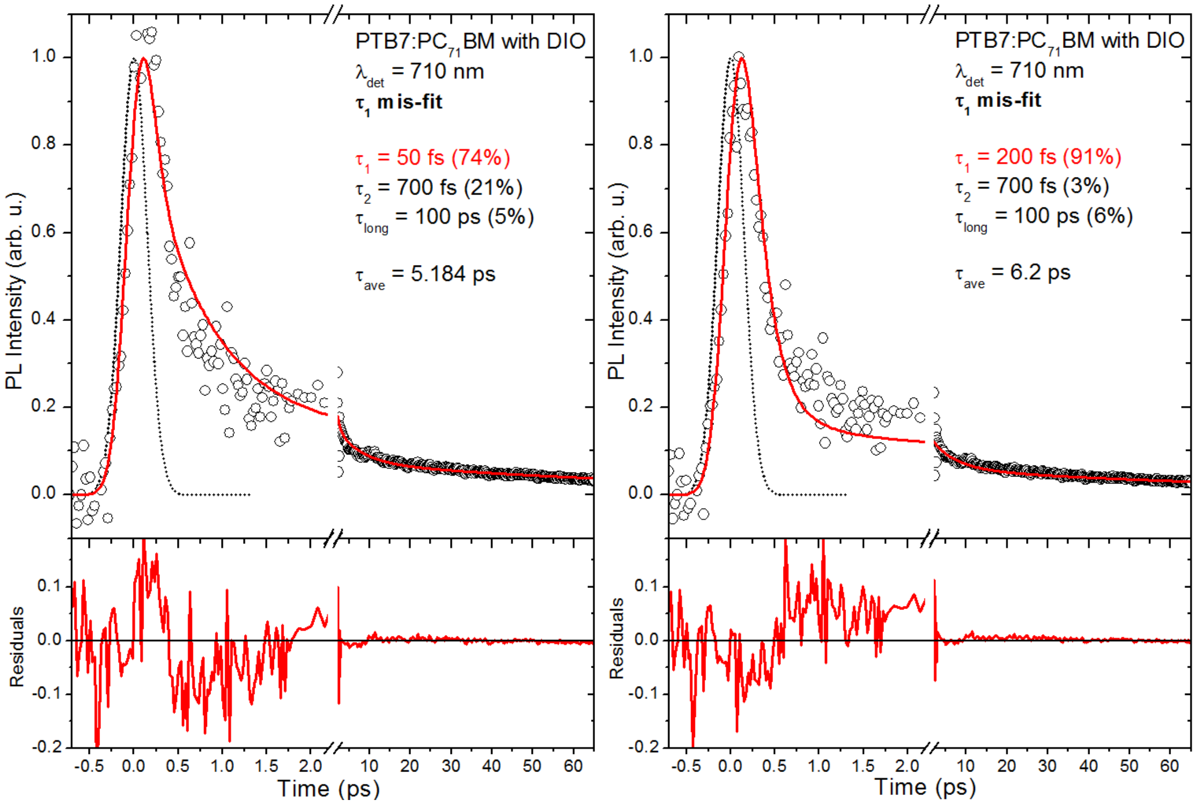

Supplementary Figure S7: Time-Resolved PL Mis-Fit: Variation of τ_1: PTB7:PC$_{71}$BM blend with DIO fluorescence decay mis-fit when the value of τ_1 is varied significantly from the best-fit value of 100 fs. It is evident that the average lifetime varies little even with significant variation of τ_1.

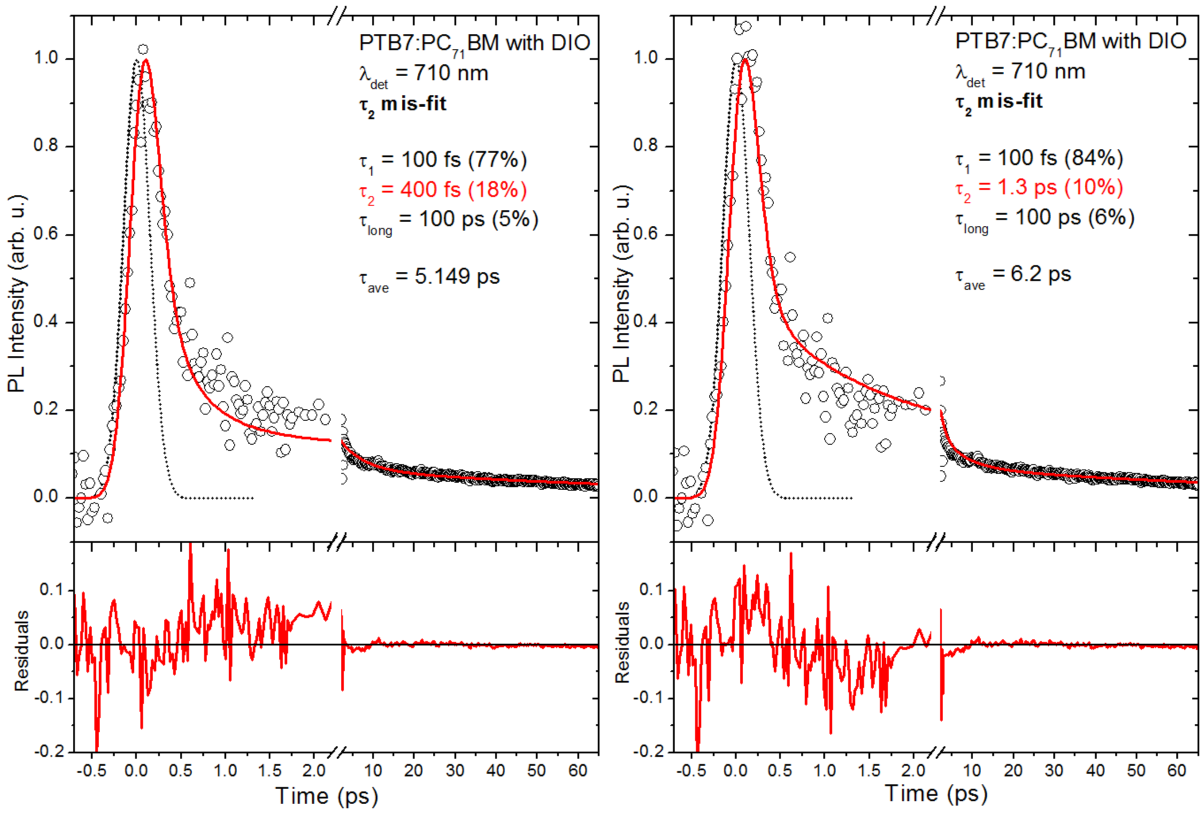

Supplementary Figure S8: Time-Resolved PL Mis-Fit: Variation of τ_2: PTB7:PC_{71}BM blend with DIO fluorescence decay mis-fit when the value of τ_2 is varied significantly from the best-fit value of 700 fs. It is evident that the average lifetime varies little even with significant variation of τ_2.

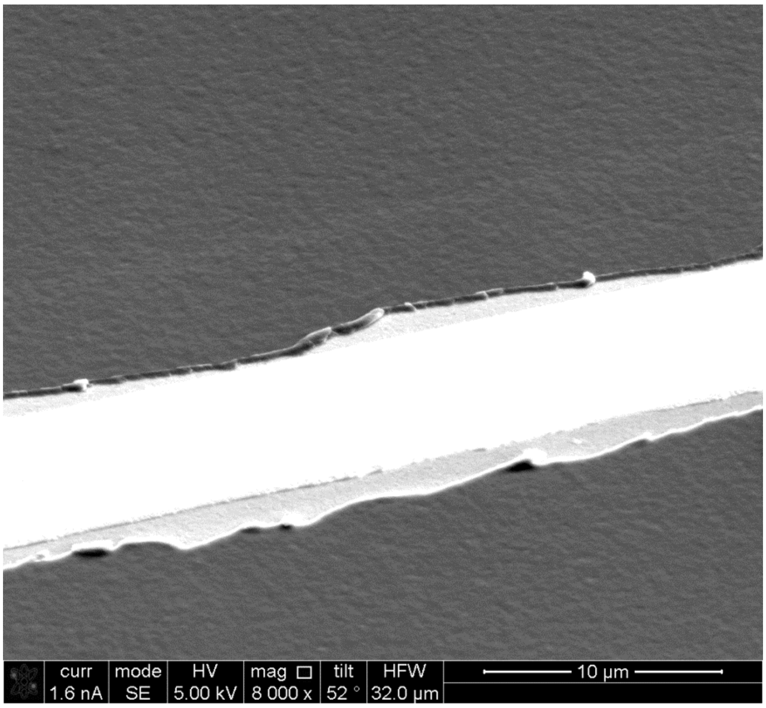

Supplementary Figure S9: Cross-Section of Blends with Additive: SEM image, taken at an angle of 52° of a score across the blend film with additive.

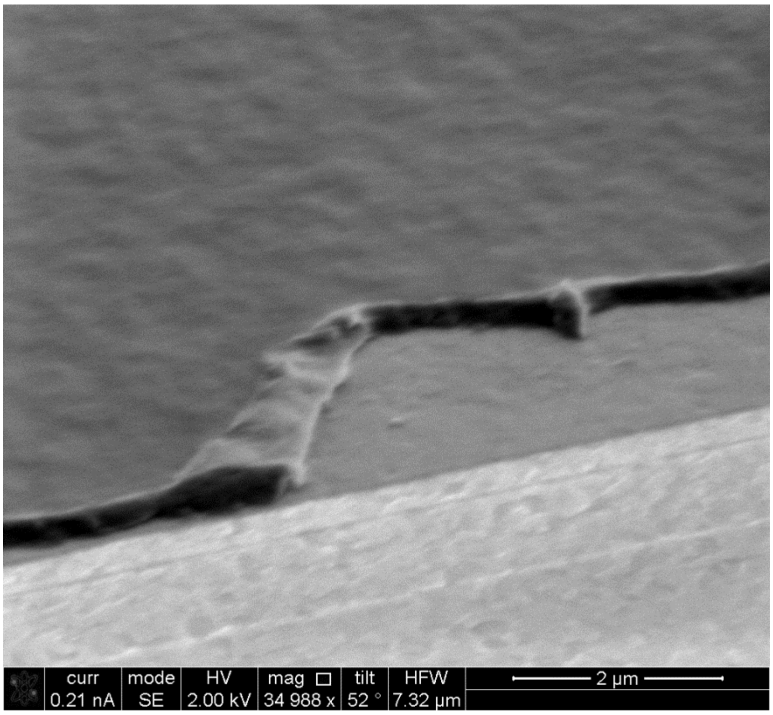

Supplementary Figure S10: Cross-Section of Blends with Additive: SEM image, taken at an angle of 52° showing the lateral uniformity of the blend with additive.

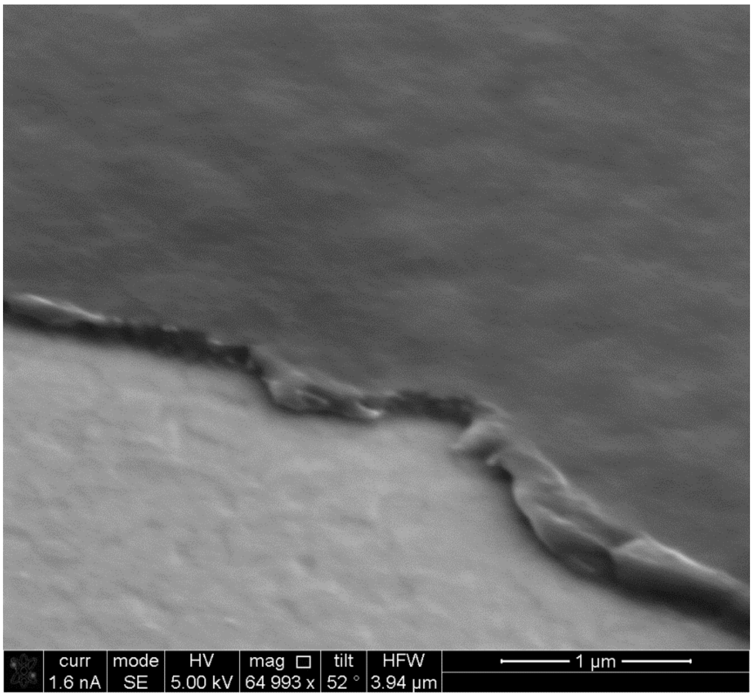

Supplementary Figure S11: Cross-Section of Blends with Additive: SEM image, taken at an angle of 52° showing the lateral uniformity of the blend with additive.

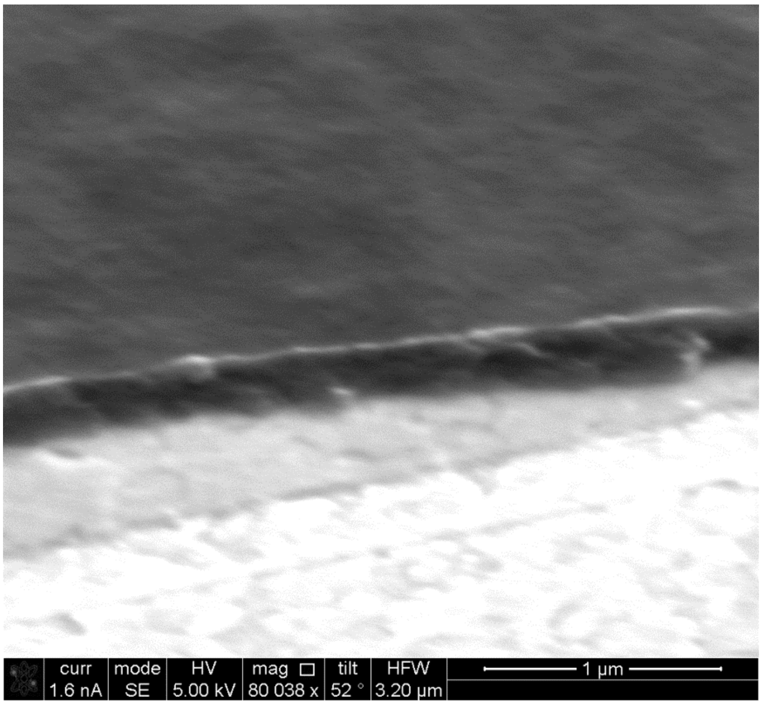

Supplementary Figure S12: Cross-Section of Blends with Additive: SEM image, taken at an angle of 52° showing the lateral uniformity of the blend with additive.

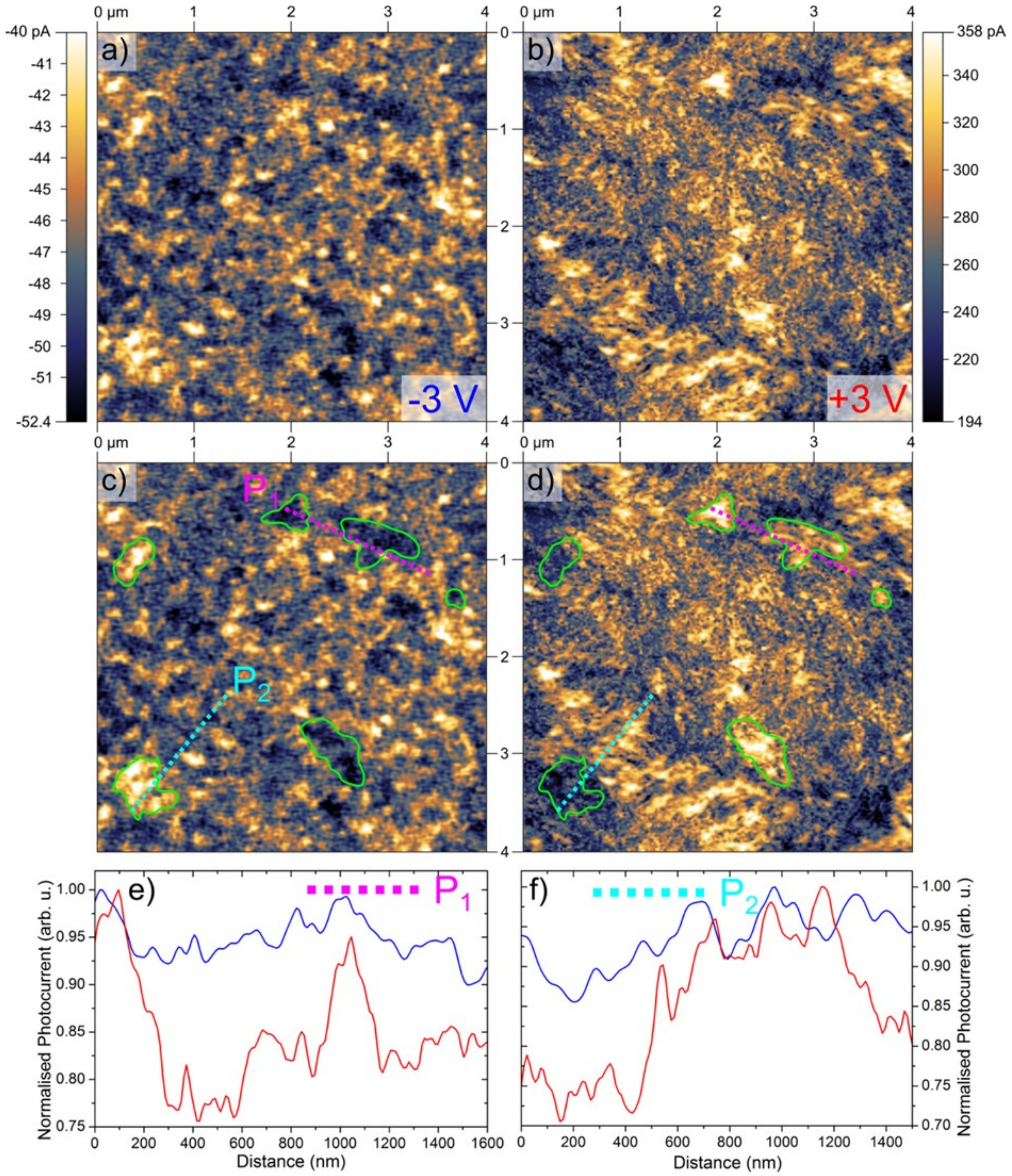

Supplementary Figure S13: Comparison of Positive and Negative Tip Bias Photocurrent Maps: Pc-AFM photocurrent maps at -3 V (**a** and **c**) and +3 V (**b** and **d**) tip bias enabling correlation of photocurrent spatial features under different bias conditions. The colour scales for the left and right maps are shown to the left and right of the panels, with a range of 10 pA at -3 V and ~ 150 pA at +3 V. Consistent with the measured photocurrent-voltage characteristics (main text, Figure 4g), the photocurrent is negative at negative tip bias, and positive at positive tip bias greater than the open circuit voltage (~0.7 V). We observe that the regions showing higher negative photocurrent at -3 V tip bias generally also show higher positive photocurrent at +3 V tip bias. These observations are annotated on the maps (**c** and **d**). The significantly reduced photocurrent at -3 V (total range 10 pA) compared to when at +3 V (total range ~150 pA) leads to a significant reduction in feature contrast, meaning that we do not observe a like-for-like inversion of the photocurrent maps, as fixed noise will affect the negative bias map far more than the positive one. These results tell us that where we record high photocurrent we see it no matter whether we have +3 or -3 V tip bias. The 670 nm light is mostly (80%) absorbed by the polymer, and so the observed photocurrent maps are dominated by the spatial position of the polymer – thus at +3 V we are extracting photoelectrons from the fullerene, but we are doing so in polymer rich regions where the photoelectrons originated as part of an exciton created 80% of the time on the polymer. In features annotated in **c** and **d** the intensities anti-correlate, i.e. negative photocurrents at negative bias correlate with positive photocurrents at positive bias. Two profiles (40 pixels wide) have been made across the photocurrent maps, and are annotated as P_1 and P_2. These profiles are shown in **e** and **f** – here the -3 V photocurrent (blue line) has been inverted to make it positive and both it and the +3 V photocurrent (red line) intensities have been normalised to enable easier comparison. General correlation can be observed between the two profiles, with a lower response to features found in negative bias.

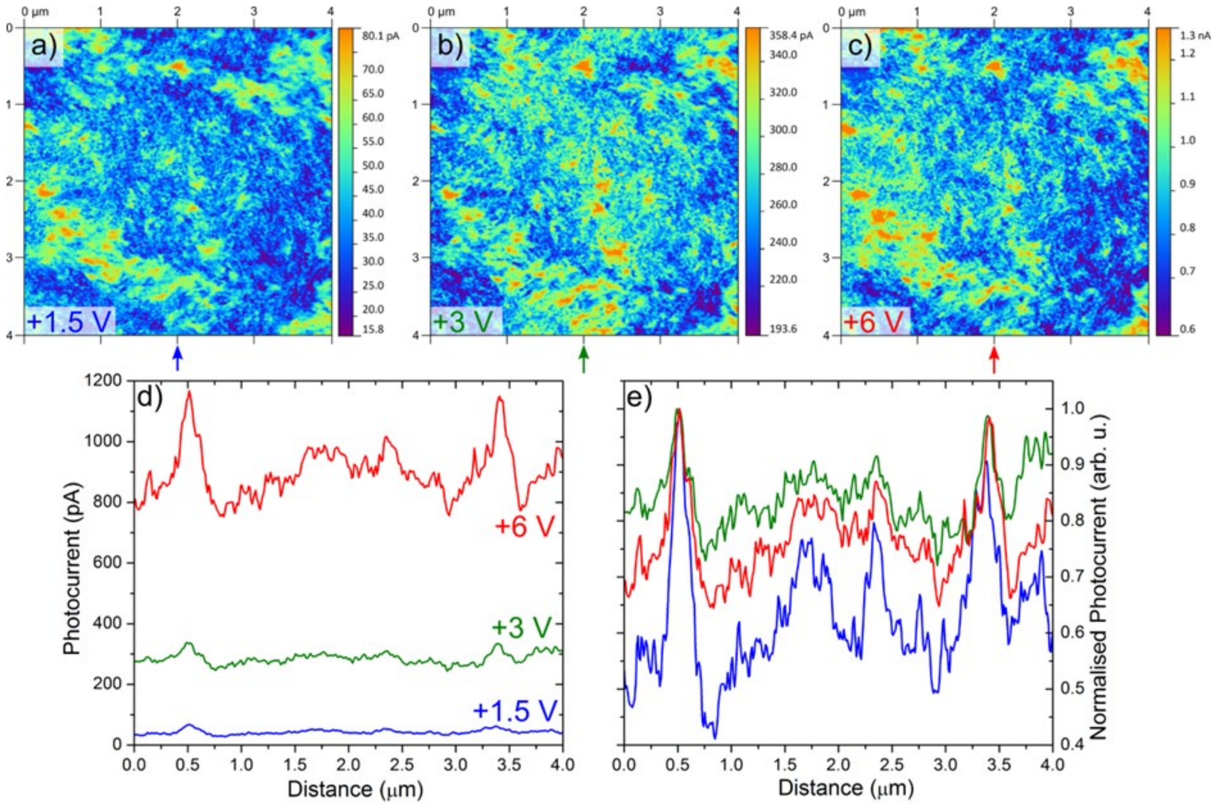

Supplementary Figure S14: Correlation in pc-AFM at Different Tip Biases: Pc-AFM photocurrent maps at +1.5 V (**a**), +3 V (**b**) and +6 V (**c**) tip bias – the colour scales for each map are shown to the right of each window. Vertical profiles 40 pixels wide are taken at x = 2 μm, as indicated with the coloured arrow below each map, and these profiles are shown in (**d**) in absolute current units from the maps as indicated, and normalised in panel (**e**). A strong degree of correlation is observed between the three tip biases.

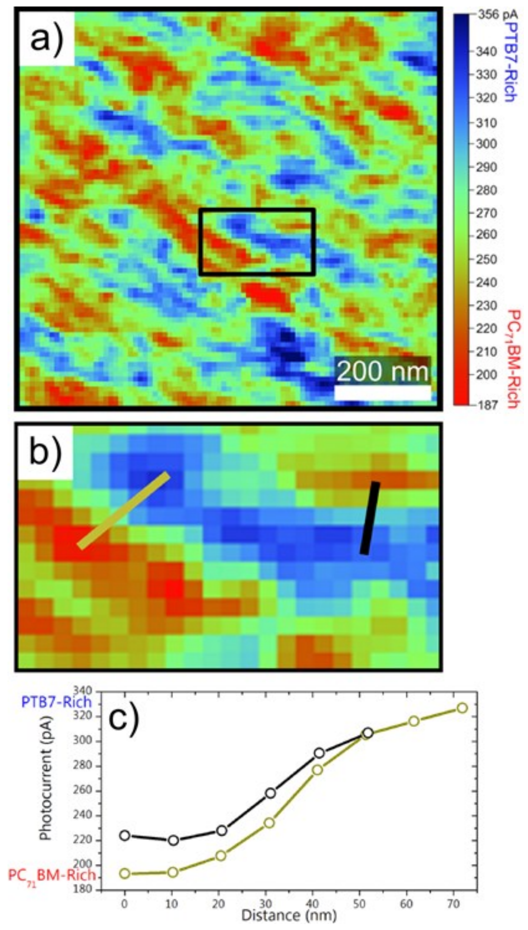

Supplementary Figure S15: Cross-Section of pc-AFM region: a) pc-AFM photocurrent map of selected region as shown in Figure 4f in the main text, the black box highlights a region shown in close-up in **b)**. Two cross-section lines are shown in the close-up, representing transitions from polymer-rich to fullerene-rich fibres. The photocurrent profile of these cross-sections is shown in **c)**, indicating that there is not a specific jump from low to high photocurrent, but rather a gradual change.

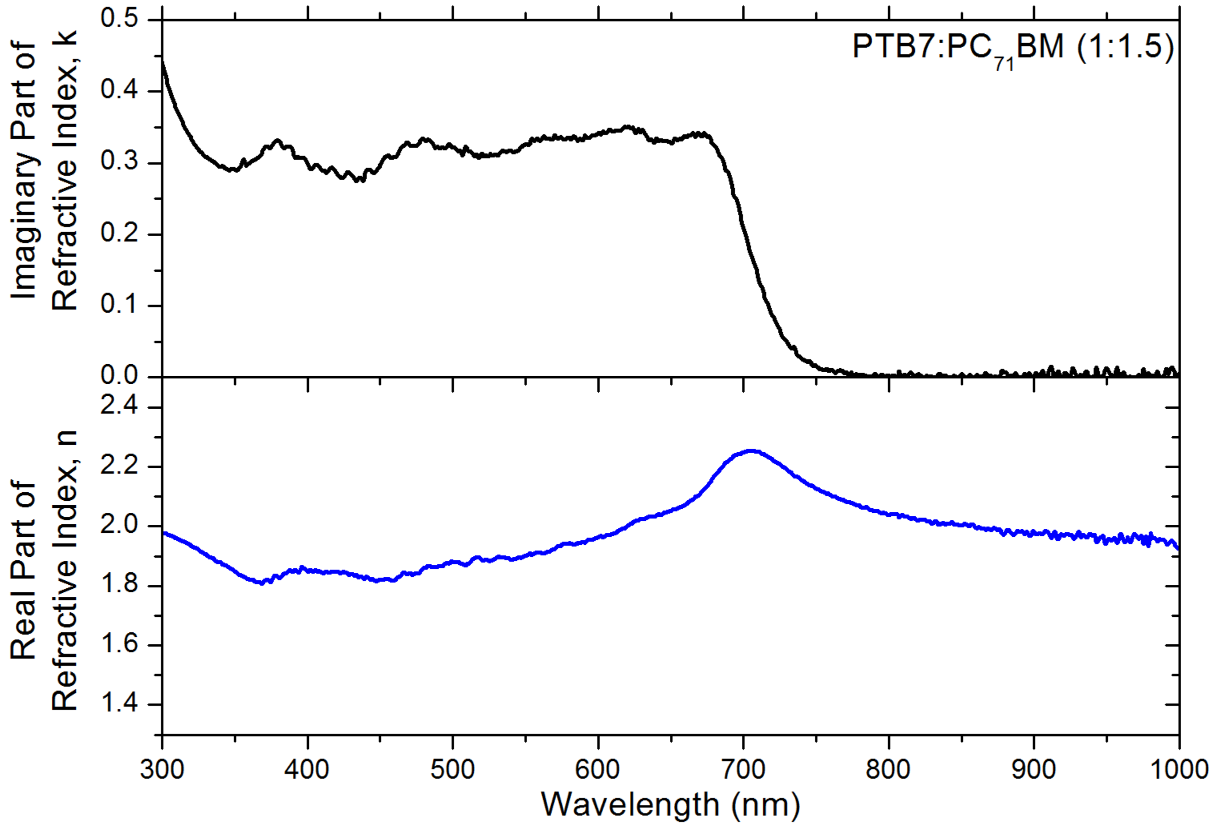

Supplementary Figure S16: Optical Constants of Active Layer from Ellipsometry: The imaginary and real part of the refractive index of the PTB7:PC_{71}BM blend, as measured with ellipsometry.
